# Supplementary material for: The Impact of Educational Intervention on Willingness to Enroll in a Clinical Trial of a Gonorrhea Vaccine
Source: Vaccines (Basel). 2023 Mar 14;11(3):648. doi: 10.3390/vaccines11030648 (PMC10053990; doi:10.3390/vaccines11030648)
Supplement: Supplementary file 1 [file vaccines-11-00648-s001.zip › vaccines-2237225-supplementary.pdf]

The Impact of Educational Intervention on Willingness to Enroll in a Clinical Trial of a Gonorrhea Vaccine

Michael Penlington [1,*], Uwe Nicolay [2] and Ilaria Galgani [3]

[1] GlaxoSmithKline Biologicals S.A., Avenue Fleming 20, 1300 Wavre, Belgium

[2] GlaxoSmithKline Vaccines GmbH, Emil-von-Behring-Straße 76, 35041 Marburg, Germany [3] GlaxoSmithKline S.p.A., Via Fiorentina 1, 53100 Siena, Italy

* Correspondence: michael.c.penlington@gsk.com

Supplementary Materials

Supplementary Tables

Table S1. Distribution of sex and ethnicity/race among age groups (*N* = 450)

Age (years)	Sex, n (%)			Ethnicity/race, n (%)			
	Male (*n* = 218)	Female (*n* = 225)	Non-binary/prefer not to say (*n* = 6)	Black/African American (*n* = 142)	White (*n* = 236)	Mixed race (*n* = 34)	Aggregation of less frequently reported ethnicities/races* (*n* = 38)
**18–24**	88 (40.4)	90 (40.0)	5 (83.3)	57 (40.1)	99 (41.9)	15 (44.1)	13 (34.2)
**25–30**	85 (39.0)	90 (40.0)	1 (16.7)	57 (40.1)	92 (39.0)	12 (35.3)	15 (39.5)
**31–49**	45 (20.6)	45 (20.0)	0	28 (19.7)	45 (19.1)	7 (20.6)	10 (26.3)

* Less frequently reported ethnicities/races = American Indian, Alaskan Native, Asian, Native Hawaiian, Pacific Islander, Hispanic, Latino, other ethnicities/races.

There was no significant association between age category and sex and age category and ethnicity/race; Chi-square $p > 0.05$.

Table S2. Number of sexual partners in the previous 6 months across sex, age, ethnicity/race, and MSM vs other (N = 450)

Sexual partners in the previous 6 months (n)	Sex, n (%)			Age (years), n (%)			Ethnicity/race, n (%)				MSM vs other, n (%)	
	Male (*n* = 218)	Female (*n* = 225)	Non-binary/ prefer not to say (*n* = 7)	18–24 (*n* = 184)	25–30 (*n* = 176)	31–49 (*n* = 90)	Black/ African American (*n* = 142)	White (*n* = 236)	Mixed race (*n* = 34)	Aggregation of less frequently reported ethnicities/ races* (*n* = 38)	MSM (*n* = 32)	Other (*n* = 418)
**0**	8 (3.7)	3 (1.3)	1 (16.7)	5 (2.7)	6 (3.4)	1 (1.1)	4 (2.8)	3 (1.3)	2 (5.9)	3 (7.9)	0	12 (2.9)
**1**	115 (52.8)	169 (75.1)	3 (50.0)	111 (60.3)	113 (64.2)	64 (71.1)	80 (56.3)	159 (67.4)	23 (67.6)	26 (68.4)	7 (21.9)	281 (67.2)
**2**	44 (20.2)	31 (13.8)	1 (16.7)	36 (19.6)	26 (14.8)	14 (15.6)	34 (23.9)	31 (13.1)	7 (20.6)	4 (10.5)	11 (34.4)	65 (15.6)
**3**	26 (11.9)	13 (5.8)	0	16 (8.7)	18 (10.2)	5 (5.6)	11 (7.7)	22 (9.3)	2 (5.9)	4 (10.5)	5 (15.6)	34 (8.1)
**4**	13 (6.0)	4 (1.8)	0	5 (2.7)	9 (5.1)	3 (3.3)	8 (5.6)	9 (3.8)	0	0	7 (21.9)	10 (2.4)
**5**	4 (1.8)	2 (0.9)	1 (16.7)	6 (3.3)	0	1 (1.1)	3 (2.1)	4 (1.7)	0	0	1 (3.1)	6 (1.4)
**≥6**	7 (3.2)	1 (0.4)	0	3 (1.6)	4 (2.3)	1 (1.1)	1 (0.7)	6 (2.5)	0	1 (2.6)	1 (3.1)	7 (1.7)
**Mean ranks**	2.53	2.27	2.50	2.48	2.36	2.30	2.40	2.42	2.26	2.34	2.66	2.38
**Prefer not to say**	1 (0.5)	2 (0.9)	0	2 (1.1)	0	1 (1.1)	1 (0.7)	2 (0.8)	0	0	0	3 (0.7)

* Less frequently reported ethnicities/races = American Indian, Alaskan Native, Asian, Native Hawaiian, Pacific Islander, Hispanic, Latino, other ethnicities/races.

MSM, men who have sex with men.

There was a statistically significant association between the number of sexual partners and sex (Kruskal-Wallis $p < 0.001$), and the number of sexual partners of MSM vs other (Kruskal-Wallis $p < 0.001$) but not between the number of sexual partners and age (Kruskal-Wallis $p = 0.358$) nor between the number of sexual partners and ethnicity/race (Kruskal-Wallis $p = 0.058$).

Table S3. Distribution of age groups, MSM versus other, and ethnicity/race in US states according to prevalence (N = 450)

Rates of reported cases/100,000 population	Age (years), n (%)			MSM vs other, n (%)		Ethnicity/race, n (%)			
	18–24 (*n* = 184)	25–30 (*n* = 176)	31-49 (*n*=90)	MSM (*n* = 32)	Other (*n* = 418)	Black/African American (*n* = 142)	White (*n* = 236)	Mixed race (*n* = 34)	Aggregation of less frequently reported ethnicities/ races* (*n* = 38)
**15–98**	4 (2.2)	4 (2.3)	1 (1.1)	0	9 (2.2)	1 (0.7)	8 (3.4)	0	0
**99–154**	42 (22.8)	44 (25.0)	17 (18.9)	8 (25.0)	95 (22.7)	30 (21.3)	57 (24.2)	6 (17.6)	10 (26.3)
**155–182**	72 (39.1)	66 (37.5)	38 (42.2)	17 (53.1)	159 (38.0)	62 (44.0)	76 (32.2)	16 (47.1)	22 (57.9)
**183–230**	38 (20.7)	40 (22.7)	19 (21.1)	5 (15.6)	92 (22.0)	31 (22.0)	55 (23.3)	7 (20.6)	4 (10.5)
**231–264**	28 (15.2)	22 (12.5)	15 (16.7)	2 (6.3)	63 (15.1)	17 (12.1)	40 (16.9)	5 (14.7)	2 (5.3)
**Mean ranks**	2.63	2.68	2.67	2.78	2.65	2.75	2.58	2.74	2.74

* Less frequently reported ethnicities/races = American Indian, Alaskan Native, Asian, Native Hawaiian, Pacific Islander, Hispanic, Latino, other ethnicities/races.

MSM, men who have sex with men.

There was a statistically significant association between ethnicity/race and State case rates (Kruskal-Wallis p = 0.025) but not with age (Kruskal-Wallis p = 0.979) nor MSM vs other (Kruskal-Wallis p = 0.326) and State case rates.

Figure S1. Survey questionnaire.

QUESTIONNAIRE

NB. Supporting text on all questions in the grey boxes is for internal use.

1. Question text:

A global pharmaceutical company seeks to understand people's attitudes to and opinions of vaccine research studies performed by pharmaceutical companies when developing vaccines. After finding out a little about you, we'll tell you more about these vaccine healthcare initiatives.

This research, which consists of a survey that will take around 15 minutes of your time today, is sponsored by the pharmaceutical company and run by InSites Consulting, an independent research company, with no promotional intent. Your participation will be confidential to InSites Consulting. Your answers will be shared with the company sponsoring the research but will not be attributed to you as an individual.

Personal information collected during the survey will include your age, gender, household size and in-come, and some health details. This personal information will be processed solely for purposes of this research study and for reporting any adverse events. All your responses will be kept confidential, anonymous. They will be stored in line with market research codes of conduct and data protection laws.

The pharmaceutical company sponsoring this survey may wish to publish the results of this research. However, anything you say or submit during this research will be anonymous and not directly attributed to you.

You can contact your panel support team for details of how the sponsor processes your personal in-formation and your rights about this information

Sometimes your answers to this survey indicate that you might have had a problem with a product or experienced some form of side effect. Product manufacturers are keen to ensure the safety of their products, and, in some cases, have legal obligations to report to local authorities any problems users may experience. We are working with a company that monitors the performance of its products in the market and the health and wellbeing of consumers who use them. If your responses to this survey indicate that you may have had such a problem, we are required to share this with the manufacturer in an anonymous format.

After satisfactorily completing this survey, you will receive credit(s) in line with Panel terms and conditions.

By clicking on 'I agree' below, you confirm that you are willing to participate in this research under the provisions described and that you consent to the sponsor using your personal information. At any time, you have the right to withhold information and/or withdraw from this research.

Answer options	Precodes	Fix	Open	Screen
I agree	1	☐	☐	☐
I do not agree	2	☐	☐	☒

2. Question text:

By providing your consent to participate in this research study, you agree to keep all information dis-closed to you about the study, including any products, services, ideas, concepts, packaging, and advertising, completely confidential. You further agree not to disclose such information to any other party, nor to attempt to take, copy, remove, print, record, or download any materials, notes, questionnaires, videos, charts or other information from this study. You also agree to use the information disclosed to you only for participating in this research study.

In addition, you acknowledge that you and others in your household do not work in the consumer or pharmaceutical healthcare product development, market research, or advertising industries.

By clicking on ‘I agree’ below, you are confirming that you agree to the provisions that have been described.

Answer options	Precodes	Fix	Open	Screen
I agree	1	☒	☐	☐
I do not agree	2	☒	☐	☒

3. Question text:
Before we get into today's survey, we have a few questions to make sure that it is relevant to you.
Which of the following best describes your sex assigned at birth?

Page break: Yes
Title: Q3
Question type: Single Select
Randomisation: None
Implementation QID: Q3 - Sex
Filter / routing information: No
Other potential instructions:

Answer options	Precodes	Fix	Open	Screen
Male<br>MAX QUOTA: n=225	1	☐	☐	☐
Female<br>MAX QUOTA: n=225	2	☐	☐	☐
Non-binary	3	☐	☐	☐
Prefer not to say	4	☐	☐	☐

4. Question text:
In which of the following states or areas do you live (as your main residence)?

Instruction text: Click on the dropdown menu and select the state or area you live in.

Answer options	Precodes	Fix	Open	Screen
Alabama	1	☐	☐	☐
Alaska	2	☐	☐	☐
Arizona	3	☐	☐	☐
Arkansas	4	☐	☐	☐
California	5	☐	☐	☐
Colorado	6	☐	☐	☐
Connecticut	7	☐	☐	☐
Delaware	8	☐	☐	☐
Florida	9	☐	☐	☐
Georgia	10	☐	☐	☐
Hawaii	11	☐	☐	☐
Idaho	12	☐	☐	☐
Illinois	13	☐	☐	☐
Indiana	14	☐	☐	☐
Iowa	15	☐	☐	☐
Kansas	16	☐	☐	☐
Kentucky	17	☐	☐	☐
Louisiana	18	☐	☐	☐
Maine	19	☐	☐	☐
Maryland	20	☐	☐	☐
Massachusetts	21	☐	☐	☐
Michigan	22	☐	☐	☐
Minnesota	23	☐	☐	☐
Mississippi	24	☐	☐	☐

Missouri	25	☐	☐	☐
Montana	26	☐	☐	☐
Nebraska	27	☐	☐	☐
Nevada	28	☐	☐	☐
New Hampshire	29	☐	☐	☐
New Jersey	30	☐	☐	☐
New Mexico	31	☐	☐	☐
New York	32	☐	☐	☐
North Carolina	33	☐	☐	☐
North Dakota	34	☐	☐	☐
Ohio	35	☐	☐	☐
Oklahoma	36	☐	☐	☐
Oregon	37	☐	☐	☐
Pennsylvania	38	☐	☐	☐
Rhode Island	39	☐	☐	☐
South Carolina	40	☐	☐	☐
South Dakota	41	☐	☐	☐
Tennessee	42	☐	☐	☐
Texas	43	☐	☐	☐
Utah	44	☐	☐	☐
Vermont	45	☐	☐	☐
Virginia	46	☐	☐	☐
Washington	47	☐	☐	☐
West Virginia	48	☐	☐	☐
Wisconsin	49	☐	☐	☐

Wyoming	50	☐	☐	☐
Washington, D.C.	51	☐	☐	☐
Puerto Rico	52	☐	☐	☐

5. Question text:
How old are you?

Answer options	Precodes	Fix	Open	Screen
Under 18<br>Where Q4=1 OR 27 SHOW<br>Under 19<br>Where Q4=24 SHOW<br>Under 21	1	☐	☐	☒
18-24<br>Where Q4=1 OR 27 SHOW<br>19-24<br>Where Q4=24 SHOW<br>21-24<br>MAX QUOTA: n=180	2	☐	☐	☐
25-30<br>MAX QUOTA: n=180	3	☐	☐	☐
31-49<br>MAX QUOTA: n=90	4	☐	☐	☐
50+	5	☐	☐	☒

6. Question text:

Approximately, what is your combined yearly household income?

Instruction text: By combined yearly household income, we mean the total amount that everyone in your household earns on a yearly basis.
We’re looking to speak to a broad range of people, so please give us your honest estimate. If you do not feel comfortable answering this, please tell us that you prefer not to say.
Please be assured that this information will not be shared with anybody else and is only to ensure we speak to people from a mix of backgrounds.

Answer options	Precodes	Fix	Open	Screen
Under $15,000	1	☐	☐	☐
$15,000 - $24,999	2	☐	☐	☐
$25,000 - $34,999	3	☐	☐	☐
$35,000 - $49,999	4	☐	☐	☐
$50,000 - $74,999	5	☐	☐	☐
$75,000 - $99,999	6	☐	☐	☐
$100,000 - $149,999	7	☐	☐	☐
$150,000 - $199,999	8	☐	☐	☐
$200,000 and over	9	☐	☐	☐
Prefer not to say	10	☐	☐	☐

7. Question text:

Today, we are looking to speak to people who identify with a wide variety of different communities. To make sure we're speaking to different people, we would now like to ask you a couple of questions that may be seen as sensitive, such as your race and ethnicity. Providing this information is completely voluntary, and you can always say that you 'Prefer not to say' if you don't feel comfortable sharing.

Do you consent to us collecting this information about you?

Answer options	Precodes	Fix	Open	Screen
Yes, I consent	1	☐	☐	☐
No, I do not consent	2	☐	☐	☒
Prefer not to say	3	☐	☐	☒

7b. **Question text:**

Do you consider yourself to be Hispanic or Latino?

Instruction text: *We are looking to speak to people who identify with a wide variety of different communities. If you are not comfortable sharing, just let us know that you prefer not to say, although we will be unable to continue with the interview at this point.*
So everyone is on the same page, by 'Hispanic or Latino', we mean a person of Cuban, Mexican, Puerto Rican, South or Central American, or other Spanish culture or origin, regardless of geographic ancestry or race.

Answer options	Precodes	Fix	Open	Screen
Yes	1	☐	☐	☐
No	2	☐	☐	☐
Prefer not to say	3	☐	☐	☒

8. Question text:

What of the following five racial designations best describes you?

Instruction text: Please tell us all that apply. We are looking to speak to people who identify with a wide variety of different communities. If you are not comfortable sharing, just let us know that you prefer not to say, although we will be unable to continue with the interview at this point.

Answer options	Precodes	Fix	Excl.	Open	Screen
American Indian or Alaskan Native<br><br><Show following text if hovered over: A person having origins in any of the original peoples of North, South, and Central America, and who maintains tribal affiliation or community attachment.>	1	☐	☐	☐	☐
Asian<br><br><Show following text if hovered over: A person having origins in any of the original peoples of the Far East, Southeast Asia or the Indian subcontinent.>	2	☐	☐	☐	☐
Black / African American<br><br><Show following text if hovered over: A person having origins of any of the Black racial groups of Africa or of Caribbean descent.>	3	☐	☐	☐	☐
Native Hawaiian or Pacific Islander<br><br><Show following text if hovered over: A person having origins in any of the original peoples of Hawaii, Guam, Samoa, or other Pacific Islands.>	4	☐	☐	☐	☐
White<br><br><Show following text if hovered over: A person having origins in any of the original peoples of Europe, the Middle East or North Africa.>	5	☐	☐	☐	☐
Other	6	☐	☐	☐	☐
Prefer not to say	7	☐	☐	☐	☒

9. Question text: Our topic today might be considered sensitive in nature, so we'd now like to ask a bit more of a personal question, to make sure we're speaking to the right audience.

As we mentioned before, your responses will be anonymised and we're not collecting any information that can personally identify you, so please give us your honest answers.

Would you consider yourself to be sexually active?

(By 'sexually active' we mean engaging in any form of sexual activity over the past 6 months)

Answer options	Precodes	Fix	Open	Screen
Yes	1	☐	☐	☐
No	2	☐	☐	☒
Prefer not to say	3	☐	☐	☒

10. Question text: How many sexual partners have you had in the last 6 months?

Answer options	Precodes	Fix	Open	Screen
0<br><br>MAX QUOTA: n=15	1	☐	☐	☐
1	2	☐	☐	☐
2	3	☐	☐	☐
3	4	☐	☐	☐
4	5	☐	☐	☐
5	6	☐	☐	☐
6+	7	☐	☐	☐
Prefer not to say<br><br>MAX QUOTA: n=15	8	☐	☐	☐

11. Question text:

Which of the following best describes your sexual orientation?

Instruction text: Please select all that apply.

Answer options	Precodes	Fix	Excl.	Open	Screen
Heterosexual (straight)	1	☐	☐	☐	☐
Gay	2	☐	☐	☐	☐
Lesbian	3	☐	☐	☐	☐
Bisexual	4	☐	☐	☐	☐
Asexual	5	☐	☐	☐	☐
Pansexual	6	☐	☐	☐	☐
Other	7	☐	☐	☐	☐
Prefer not to say	98	☒	☐	☐	☐

12. Question text:

Thinking about your attitude towards healthcare more broadly…

Which of the following best describes your feelings towards vaccines?

Answer options	Precodes	Fix	Open	Screen
I have had vaccines before and I would be open to having them again	1	☐	☐	☐
I have had vaccines before, but I would not be open to having them again	2	☐	☐	☐
I haven't had vaccines before, but I would be open to having them	3	☐	☐	☐
I haven't had vaccines before and I would not be open to having them	4	☐	☐	☐

13. Question text:

Which of the following reasons fit you best in terms of why you are not open to having future vaccines?

Answer options	Precodes	Fix	Open	Screen
I was vaccinated as a child, it was not my choice to have past vaccines	1	☐	☐	☐
I am afraid of needles	2	☐	☐	☐
I don't think vaccines are safe	3	☐	☐	☐
I think vaccines cause more problems than they solve	4	☐	☐	☐
I am afraid of side effects	5	☐	☐	☐
I don't feel I need a vaccine, I live a healthy lifestyle and have a good immune system	6	☐	☐	☐
I don't think I'm at risk of getting seriously ill	7	☐	☐	☐
I haven't seen any information that would convince me that getting future vaccines is safe and would benefit me	8	☐	☐	☐
I can't afford to get vaccinated, the process costs too much	9	☐	☐	☐
I don't trust the government/ pharmaceutical companies	10	☐	☐	☐
I don't have time to get vaccinated	11	☐	☐	☐
Another reason (please specify)	12	☒	☒	☐

Info Text:
Thank you for answering those up-front questions. We're pleased to say that you are exactly who we want to answer the rest of our questions, which, as you may have guessed, primarily focus on vaccine clinical studies.

When you're ready to see the first questions, click the 'Continue' below to continue.

14. Question text: Which of the below best describes your general understanding awareness and knowledge of clinical research studies?

By 'clinical study', we mean a research study that looks to understand how a medicine works or how safe it is.

Please note, we are not asking you to take part in a clinical research study, nor will we, we're just interested in understanding your opinions.

Answer options	Precodes	Fix	Open	Screen
I have never heard of a clinical research study before	1	☐	☐	☐
I have heard of clinical research studies, but I don't know anything / only know very little about them	2	☐	☐	☐
I have heard of clinical research studies and know a little about them	3	☐	☐	☐
I have heard of clinical research studies and know a fair amount about them	4	☐	☐	☐
I have heard of clinical research studies and know a lot about them	5	☐	☐	☐

15. Question text:

Thinking about what you know about clinical studies…

On a scale of 1 – 5, where 1 = ‘I would be very unlikely to consider taking part’ and 5 = ‘I would be very likely to consider taking part’, how likely would you be to take part in a clinical research study related to vaccines?

Instruction text: By ‘vaccine clinical study’, we mean a clinical research study that looks to understand how a vaccine works or how safe it is.
Please note, we are not asking you to take part in a clinical research study, nor will we, we’re just interested in understanding your opinions.

Answer options	Precodes	Fix	Open	Screen
1 – I would be very unlikely to consider taking part	1	☐	☐	☐
2	2	☐	☐	☐
3	3	☐	☐	☐
4	4	☐	☐	☐
5 – I would be very likely to consider taking part	5	☐	☐	☐

16. Question text:

<IF Q15 = 1 and 2 (i.e. are unlikely to take part)> You said you would be unlikely to take part in a vaccine clinical study. Here are some reasons that other people have told us might put them off or make it difficult for them to take part in vaccine clinical studies.

<IF Q15 = 3 (i.e. neither likely nor unlikely)> You said you would be neither likely nor unlikely to take part in a vaccine clinical study. Here are some reasons that other people have told us might put them off or make them uncertain about taking part in vaccine clinical studies.

For each of these reasons, please tell us whether they apply to you, and whether they are concerns you would currently have if asked to take part in a vaccine clinical study.

<u>Click on the tick or swipe right</u> if you think that each reason would put you off or make it difficult for you to take part in a vaccine clinical study.

<u>Click on the cross or swipe left</u> if you think that each reason would <u>not</u> put you off or would <u>not</u> make it difficult for you to take part in a vaccine clinical study.

Instruction text:
Are you sure this would put you off or make it difficult for you to take part in a vaccine clinical study? / Are you sure this would <u>not</u> put you off or make it difficult for you to take part in a vaccine clinical study?
Dragging a statement to the left means you think this reason would <u>not</u> put you off or make it difficult for you to take part in a vaccine clinical study. / Dragging a statement to the right means think this reason <u>would</u> put you off or make it difficult for you to take part in a vaccine clinical study.

Answer options	Precodes	Fix	Excl.	Open	Screen
I do not have enough information about the personal risks and benefits of the vaccine being studied	1	☐	☐	☐	☐
I do not have enough information about the social benefits of the vaccine being studied	2	☐	☐	☐	☐
I do not know enough about how the clinical study works and what to do when participating	3	☐	☐	☐	☐
I do not know enough about the disease area covered by the vaccine	4	☐	☐	☐	☐
The vaccine gives immediate side effects, like pain at injection site, headache, and fever	5	☐	☐	☐	☐
The vaccine causes long-term health problems	6	☐	☐	☐	☐
The vaccine might worsen my other health conditions	7	☐	☐	☐	☐
The vaccine does not work well	8	☐	☐	☐	☐
I do not know if I receive the vaccine or a placebo	9	☐	☐	☐	☐
I feel like a human guinea pig when testing the vaccine	10	☐	☐	☐	☐
I do not trust vaccines and / or vaccine research	11	☐	☐	☐	☐
I do not trust pharmaceutical companies	12	☐	☐	☐	☐
People in my community have been mistreated in clinical studies before	13	☐	☐	☐	☐
People might judge me if I participate in a vaccine study	14	☐	☐	☐	☐
I must share private medical data when I participate	15	☐	☐	☐	☐
My data / genetic materials / samples may be used in ways I do not agree with	16	☐	☐	☐	☐
I do not get paid for participating in the study	17	☐	☐	☐	☐
I do not get paid for travel expenses to the study site	18	☐	☐	☐	☐
Too many visits to the study site interfere with my life, work, or childcare	19	☐	☐	☐	☐

17. **Question text:** You said you would be likely to take part in a vaccine clinical study.

For what reasons would you be likely to take part in a vaccine clinical study?

Instruction text: Please be as detailed as you can.

18. **Question text:** You said you would be likely to take part in a vaccine clinical study. Here are some reasons that other people have told us made them consider taking part in vaccine clinical studies.

For each of these reasons, please tell us whether they apply to you, and whether they are motivations you would currently have if asked to take part in a vaccine clinical study.

Click on the tick or swipe right if you think that each reason would make you want to take part in a vaccine clinical study.

Click on the cross or swipe left if you think that each reason would not be a key reason for your want to take part in a vaccine clinical study.

Instruction text:
Are you sure?
Dragging a statement to the left means you think this reason would not be a key reason for your want to take part in a vaccine clinical study. / Dragging a statement to the right means think this reason would make you want to take part in a vaccine clinical study.

Answer options	Precodes	Fix	Excl.	Open	Screen
My doctor discusses the clinical study with me and answers my questions	1	☐	☐	☐	☐
A healthcare professional answers my questions before the study starts	2	☐	☐	☐	☐
My doctor runs the clinical study	3	☐	☐	☐	☐
Researchers are from my community	4	☐	☐	☐	☐
The study takes place at convenient nearby locations, such as a hospital or doctor’s office	5	☐	☐	☐	☐
I am given more information about the disease being studied	6	☐	☐	☐	☐
I am given more information about the vaccine being studied	7	☐	☐	☐	☐
Participants from previous clinical studies speak to me about their experience	8	☐	☐	☐	☐
I can be treated / vaccinated for a disease before other people	9	☐	☐	☐	☐
I can prevent other people from getting sick	10	☐	☐	☐	☐
I am at risk of getting the disease or a more severe form of the disease the trial is studying	11	☐	☐	☐	☐
I receive free health insurance, healthcare assessments and care as part of the clinical study	12	☐	☐	☐	☐
I receive immediate medical care after experiencing vaccine side effects	13	☐	☐	☐	☐
I am compensated at each visit to thank me for participating	14	☐	☐	☐	☐
I can participate during convenient times (outside of my working hours or on the weekend)	15	☐	☐	☐	☐
Childcare facilities for my children are available at study sites	16	☐	☐	☐	☐
I receive transport to and from study visits, or I am reimbursed for transport costs	17	☐	☐	☐	☐
I get the study vaccine and visits at home	18	☐	☐	☐	☐
Study participation is made special: it takes place in a good hospital	19	☐	☐	☐	☐

19. Question text: If you were invited to take part in a vaccine clinical research study, what information, if any, would you want to know before deciding whether or not to take part?

As a reminder, by 'vaccine clinical study', we mean a research study that looks to understand how a vaccine works or how safe it is.

Please note, we are not asking you to take part in a clinical research study, nor will we, we're just interested in understanding your opinions.

Instruction text:

20. Question text: Which of the following areas would impact your likelihood to take part in a vaccine clinical study?

Instruction text: Place each statement into the buckets that apply – you can either click on the bucket to put the statement onscreen into it, or you can drag the statement into the bucket – it's up to you!

Answer options	Precodes	Fix	Open
Being paid for my time	1	☐	☐
Having my expenses paid, such as travel, parking or child-care	2	☐	☐
Receiving healthcare cover/ insurance	3	☐	☐
How much time it will take to travel back and forth	4	☐	☐
How long I will have to participate in the study (time between my first and last study visits)	5	☐	☐
Information on the disease area the vaccine is for, and why it is important	6	☐	☐
Information on how the vaccines can help society	7	☐	☐
Information on the safety of the vaccine/ side effects	8	☐	☐
Information on how the vaccine might help me personally	9	☐	☐
Details on the visits, blood draws, or any tests I have to have during the study	10	☐	☐
The visit schedule so I could see if it causes problems with my work hours	11	☐	☐
I would want to know something else (please specify)	12	☐	☐

Scale options	Precodes	Fix
1 – This would be essential to know, without understanding this I would not join the study	1	☐
2 – This would be good to know, as additional information I would like about the study/ my participation	2	☐
3 – This does not interest me, and would not impact my participation choice	3	☐

1. **Question text:** You mentioned that you would like to know something else that might impact your likelihood **to take part in a vaccine clinical study. Please tell us in detail what you would like to know**

Instruction text:

21. **Question text: You mentioned these factors** <INSERT ANSWERS CHOSEN WITH SCALE 1 AT Q20> **would have the biggest impact on your decision to take part in a vaccine clinical study. Why would they be particularly important to you?**

Instruction text:

22. Question text: Who do you trust most to provide healthcare information?

Instruction text: Select the top 3 that apply

Answer options	Precodes	Fix	Excl.	Open	Screen
My doctor	1	☐	☐	☐	☐
My family	2	☐	☐	☐	☐
My friends	3	☐	☐	☐	☐
Work colleagues	4	☐	☐	☐	☐
Federal government / federal public health agencies (CDC or NIH)	5	☐	☐	☐	☐
My state government and its health agencies	6	☐	☐	☐	☐
Healthcare websites (Mayo, Cleveland, or WHO)	7	☐	☐	☐	☐
Online blogs	8	☐	☐	☐	☐
Social media posts on Facebook, Twitter, or other platforms	9	☐	☐	☐	☐
Religious or faith leaders in my community (priests, vicars, or imams)	10	☐	☐	☐	☐
Members of my church/mosque	11	☐	☐	☐	☐
Pharmaceutical companies	12	☐	☐	☐	☐

23. Question text: Think of a recent healthcare decision you had to make..

Where did you go to get information that helped you make your decision?

Instruction text: Select the top 3 that apply

Answer options	Precodes	Fix	Excl.	Open	Screen
My family doctor	1	☐	☐	☐	☐
My nurse	2	☐	☐	☐	☐
My family	3	☐	☐	☐	☐
My friends	4	☐	☐	☐	☐
A specialised doctor	5	☐	☐	☐	☐
A pharmacist	6	☐	☐	☐	☐
An online search	7	☐	☐	☐	☐
Social media (such as Twitter, Facebook etc)	8	☐	☐	☐	☐
An online blog	9	☐	☐	☐	☐
The CDC website	10	☐	☐	☐	☐
A medical website (e.g. Mayo Clinic)	11	☐	☐	☐	☐
Other (please specify)	12	☐	☐	☒	☐

Info Text: For these next few questions, we would like you to imagine a situation where you've been invited to join a clinical study for a vaccine…

Again, please note, we are not asking you to take part in a clinical research study, nor will we, we're just interested in understanding your opinions.

24. Question text: How useful would you find the following ways of receiving information regarding a hypothetical vaccine clinical study?

Instruction text: Please rate on the scale, where 1 is not at all useful, and 5 is very useful

Answer options	Precodes	Fix	Excl.	Open	Screen
Paper handout (e.g. a flyer, a brochure, etc.)	1	☐	☐	☐	☐
A conversation with the research team (for example, a scientist, doctor, nurse)	2	☐	☐	☐	☐
A conversation with my usual doctor or nurse	3	☐	☐	☐	☐
An email from the research team	4	☐	☐	☐	☐
An email from my usual doctor or nurse	5	☐	☐	☐	☐
An informative website specifically for that study, and the participants	6	☐	☐	☐	☐
Information in my preferred non – English language	7	☐	☐	☐	☐
From my community leaders	8	☐	☐	☐	☐
From government announcements	9	☐	☐	☐	☐
From social media	10	☐	☐	☐	☐
QR cell phone scan codes codes in schools, health centres etc	11	☐	☐	☐	☐
Posters in my local club (night clubs, gym, sports club)	12	☐	☐	☐	☐
Other (please tell us what)	13	☐	☐	☐	☐

Scale options	Precodes	Fix
1 – Not at all useful	1	☐
2	2	☐
3	3	☐
4	4	☐
5 – Very useful	5	☐

25. **Question text:** Imagine your Primary Doctor mentioned that a vaccine clinical study that was relevant to you was taking place soon…

Please rank the following sources in order of which ones you would most like to receive information about the vaccine clinical study from, in order to find out more about it.

Instruction text: Click or drag each item into a rank position. To do this, just click on the source you would most like to find out information from and drag it into first place, followed by the source you would next most like to find out information from, and so on, until you have ranked them all.

Answer options	Precodes	Fix
The clinical study team (for example, a scientist, doctor or nurse)	1	☐
A specialist Healthcare Professional(s) linked to the condition which the vaccine is focused on	2	☐
Your usual General Practitioner / Primary Care Physician	3	☐
The manufacturer of the vaccine being tested in the clinical study	4	☐

Scale options	Precodes	Fix
1	1	☐
2	2	☐
3	3	☐

Info Text: Thanks for answering those questions about vaccines clinical studies generally. We'd now like to focus on some specific diseases / conditions, which relate to a question we asked you earlier.

As before, we know these are personal questions and as a reminder, all your answers are confidential and you can never be individually identified from them, we're just keen to understand your perspective, so please answer as honestly as you can.

Just click 'Continue' to answer our remaining questions about this specific disease area…

26. Question text: Which, if any, of the following diseases / conditions have you heard of?

Instruction text: Select all that apply

Answer options	Precodes	Fix	Excl.	Open	Screen
Chlamydia	1	☐	☐	☐	☐
Genital herpes	2	☐	☐	☐	☐
Gonorrhea or clap	3	☐	☐	☐	☐
Pubic lice or crabs	4	☐	☐	☐	☐
Syphilis	5	☐	☐	☐	☐
HIV	6	☐	☐	☐	☐
HPV	7	☐	☐	☐	☐
Genital warts	8	☐	☐	☐	☐
I have heard of all of these	98	☒	☒	☐	☐
I have heard of none of these	99	☒	☒	☐	☐

27. Question text: How much do you believe you know about gonorrhea?

Instruction text: Select one

Answer options	Precodes	Fix	Open	Screen
I think I have heard of gonorrhea, but I'm not sure what it is	1	☐	☐	☐
I have definitely heard of gonorrhea, but I'm not sure what it is	2	☐	☐	☐
I have heard of gonorrhea and I think I know a little about it	3	☐	☐	☐
I have heard of gonorrhea and think I know a reasonable amount about it	4	☐	☐	☐
I have heard of gonorrhea and think I know a lot about it	5	☐	☐	☐

28. **Question text:** You mentioned you think you know some information about gonorrhea…

Where did you get your information about gonorrhea from?

Instruction text: Select all that apply for each option

Answer options	Precodes	Fix	Excl.	Open	Screen
My usual doctor	1	☐	☐	☐	☐
A sexual health worker (e.g. at a clinic)	2	☐	☐	☐	☐
A nurse	3	☐	☐	☐	☐
A pharmacist	4	☐	☐	☐	☐
An official medical website (e.g. WebMD, CDC, etc.)	5	☐	☐	☐	☐
Another website (please tell us what)	6	☐	☐	☒	☐
A family member	7	☐	☐	☐	☐
A friend	8	☐	☐	☐	☐
My partner / a previous partner	9	☐	☐	☐	☐
A health information leaflet / brochure / campaign	10	☐	☐	☐	☐
Others at school / college / university	11	☐	☐	☐	☐
My school / college nurse	12	☐	☐	☐	☐
Other (please tell us where)	95	☐	☐	☒	☐

29. **Question text:** For these next few questions, we would like you to imagine that as part of a routine visit, your doctor or nurse tells you that there is a clinical study for a vaccine that might protect against gonorrhea and they're looking for people in your age group to take part.

Please note, you would not need to have gonorrhea to take part in this imaginary clinical study!

How likely would you be to sign up for a gonorrhea vaccine clinical study?

Instruction text: Select one

Answer options	Precodes	Fix	Open	Screen
1 – I would be very unlikely to consider taking part	1	☐	☐	☐
2 – I would be quite unlikely to consider taking part	2	☐	☐	☐
3 – I would be neither likely nor unlikely to consider taking part	3	☐	☐	☐
4 – I would be quite likely to consider taking part	4	☐	☐	☐
5 – I would be very likely to consider taking part	5	☐	☐	☐

30. **Question text: You said you would be unlikely to consider taking part in a gonorrhea vaccine clinical study.**

What is it that makes you unlikely to take part?

Instruction text:

Answer options	Precodes	Fix	Excl.	Open	Screen
I do not have enough information about the personal risks and benefits of the gonorrhea vaccine	1	☐	☐	☐	☐
I do not have enough information about the social benefits of the gonorrhea vaccine	2	☐	☐	☐	☐
I do not know enough about how the gonorrhea clinical study works and what to do when participating	3	☐	☐	☐	☐
I do not know enough about gonorrhea	4	☐	☐	☐	☐
The gonorrhea vaccine gives immediate side effects, like pain at injection site, headache, and fever	5	☐	☐	☐	☐
The gonorrhea vaccine causes long-term health problems	6	☐	☐	☐	☐
The gonorrhea vaccine might worsen my other health conditions	7	☐	☐	☐	☐
The gonorrhea vaccine does not work well	8	☐	☐	☐	☐
I do not know if I receive the gonorrhea vaccine or a placebo	9	☐	☐	☐	☐
I feel like a human guinea pig when testing the gonorrhea vaccine	10	☐	☐	☐	☐
I do not trust vaccines and / or vaccine research	11	☐	☐	☐	☐
I do not trust pharmaceutical companies	12	☐	☐	☐	☐
People in my community have been mistreated in clinical studies before	13	☐	☐	☐	☐
People might judge me if I participate in a gonorrhea vaccine study	14	☐	☐	☐	☐
I must share private medical data when I participate	15	☐	☐	☐	☐
My data / genetic materials / samples may be used in ways I do not agree with	16	☐	☐	☐	☐
I do not get paid for participating in the gonorrhea study	17	☐	☐	☐	☐
I do not get paid for travel expenses to the gonorrhea study site	18	☐	☐	☐	☐
Too many visits to the gonorrhea study site interfere with my life, work, or childcare	19	☐	☐	☐	☐

31. Question text: Are there any other reasons that make you unlikely to take part in a gonorrhea vaccine clinical study?

Instruction text: I*f you have multiple reasons, please list them all in the below box*

Answer options	Precodes	Fix	Screen
No	1	Fix	☐

Info Text: Thank you very much for offering your thoughts. Here is some more information about gonorrhea in the United States.

- The Center of Disease Control and Prevention estimates that on any given day in 2018, 1 in 5 people had a sexually transmitted infection or disease, also called an STI or STD
- In 2018, there were an estimated 1.6 million new cases of gonorrhea in the US
- Gonorrhea is the second most common STD
- You can get gonorrhea by vaginal, anal, or oral sex without a condom with a partner who has gonorrhea
- Gonorrhea might have no symptoms, but it can cause serious health problems, even without symptoms
- Gonorrhea can cause infertility
- Women can pass gonorrhea to their baby during childbirth
- The usual treatment for gonorrhea is antibiotic injection, usually into the glute (buttock) muscle
- Gonorrhea may make you more vulnerable to HIV infection

32. **Question text:** Now, having had some information about the disease, if as part of a routine visit, your doctor or nurse tells you that there is a clinical study for a vaccine that might protect against gonorrhea and they're looking for people in your age group to take part…

How likely would you be to sign up for a gonorrhea vaccine clinical study?

(Please note, you would not need to have gonorrhea to take part in this imaginary clinical study!)

Instruction text: Select one

Answer options	Precodes	Fix	Open	Screen
1 – I would be very unlikely to consider taking part	1	☐	☐	☐
2 – I would be quite unlikely to consider taking part	2	☐	☐	☐
3 – I would be neither likely nor unlikely to consider taking part	3	☐	☐	☐
4 – I would be quite likely to consider taking part	4	☐	☐	☐
5 – I would be very likely to consider taking part	5	☐	☐	☐

33. **Question text: You said you would be likely to consider taking part in a gonorrhea vaccine clinical study.**

What is it that makes you likely to take part?

Instruction text:

Answer options	Precodes	Fix	Excl.	Open	Screen
My doctor discusses the gonorrhea clinical study with me and answers my questions	1	☐	☐	☐	☐
A healthcare professional answers my questions before the gonorrhea study starts	2	☐	☐	☐	☐
My doctor runs the gonorrhea clinical study	3	☐	☐	☐	☐
Gonorrhea researchers are from my community	4	☐	☐	☐	☐
The gonorrhea study takes place at a convenient, nearby location (a hospital or doctor's office)	5	☐	☐	☐	☐
I am given more information about the disease caused by gonorrhea	6	☐	☐	☐	☐
I am given more information about the gonorrhea vaccine	7	☐	☐	☐	☐
Participants from previous gonorrhea clinical studies speak to me about their experience	8	☐	☐	☐	☐
I can be treated / vaccinated for gonorrhea before other people	9	☐	☐	☐	☐
I can prevent other people from getting gonorrhea	10	☐	☐	☐	☐
I am at risk of getting gonorrhea or a severe form of its disease	11	☐	☐	☐	☐
I receive free health insurance, healthcare assessments and care as part of the gonorrhea study	12	☐	☐	☐	☐
I receive immediate medical care after experiencing gonorrhea vaccine side effects	13	☐	☐	☐	☐
I am compensated at each visit to thank me for participating in the gonorrhea study	14	☐	☐	☐	☐
I can participate during convenient times (outside of my working hours or on the weekend)	15	☐	☐	☐	☐
Childcare facilities for my children are available at gonorrhea study sites	16	☐	☐	☐	☐
I receive transport to and from gonorrhea study visits or I am reimbursed for transport costs	17	☐	☐	☐	☐
I get the gonorrhea vaccine and visits at home	18	☐	☐	☐	☐
Gonorrhea study participation is made special: it takes place in a good hospital	19	☐	☐	☐	☐

34. Question text: What further information would you need to know about gonorrhea for you to decide whether or not you would wish to take part in a vaccine study for this area?

Instruction text: Select all that apply for each option

Answer options	Precodes	Fix	Excl.	Open	Screen
How many people have gonorrhea in your area / the country you live in	1	☐	☐	☐	☐
What symptoms people get with gonorrhea	2	☐	☐	☐	☐
What emotional impact living with gonorrhea has on people	3	☐	☐	☐	☐
The long term health risks of gonorrhea	4	☐	☐	☐	☐
How many people die (if any) from gonorrhea each year	5	☐	☐	☐	☐
Information about my own risk of getting gonorrhoea	6	☐	☐	☐	☐
Other (please tell us what)	7	☐	☐	☒	☐
Nothing	8	☐	☒	☐	☐

35. Question text: Imagine you were taking part in the gonorrhea study…

Is there anything further about the disease area or clinical study that you would want to know?

Instruction text:

36. Question text:

Thanks for all your answers so far! We now have a few final questions to ensure we're speaking to a range of people. These final questions are completely optional, so just let us know you don't want to share this information by pressing 'Prefer not to say'.

What is the highest level of education that you have achieved?

Answer options	Precodes	Fix	Open	Screen
Less than a high school degree	1	☐	☐	☐
High school graduate	2	☐	☐	☐
Some college education	3	☐	☐	☐
College graduate	4	☐	☐	☐
Education after college graduate	5	☐	☐	☐
Other (please tell us what)	6	☒	☒	☐
Prefer not to say	7	☒	☐	☐

37. Question text:

Which of the following best describes your insurance status?

Answer options	Precodes	Fix	Open	Screen
I have health insurance	1	☐	☐	☐
I do not have health insurance	2	☐	☐	☐
I don't know	3	☐	☐	☐

38. Question text:

Which of the following statements best describes where you live?

Answer options	Precodes	Fix	Open	Screen
An urban area	1	☐	☐	☐
A suburban area	2	☐	☐	☐
A rural area	3	☐	☐	☐

Info Text: Thank you so much for your time today – you have reached the end of the survey!
